# Sustained-Release Intra-Articular Drug Delivery: PLGA Systems in Clinical Context and Evolving Strategies

**DOI:** 10.3390/pharmaceutics17101350

**Published:** 2025-10-20

**Authors:** Jun Woo Lee, Ji Ho Park, Geon Woo Yu, Jae Won You, Min Ji Han, Myung Joo Kang, Myoung Jin Ho

**Affiliations:** College of Pharmacy, Dankook University, 119 Dandae-ro, Dongnam-gu, Cheonan 330-714, Chungnam, Republic of Korea; junwoolee5776@gmail.com (J.W.L.); jihop0214@gmail.com (J.H.P.); dbrjsdn0311@naver.com (G.W.Y.); jayyou5094@gmail.com (J.W.Y.); mjhan256@gmail.com (M.J.H.); kangmj@dankook.ac.kr (M.J.K.)

**Keywords:** intra-articular drug delivery, PLGA microspheres, sustained release, long-acting injectable, corticosteroid depot, acidic microclimate (PLGA), biologic/peptide stabilization

## Abstract

Poly(lactic-co-glycolic acid) (PLGA) sustained-release systems for intra-articular (IA) delivery aim to extend joint residence time and reduce the reinjection frequency of conventional IA therapies. This review synthesizes current understanding of PLGA degradation, the acidic microenvironment inside degrading microspheres, and release behavior in joints, and surveys clinical experience with extended-release corticosteroid depots alongside emerging platforms for nonsteroidal and biologic agents. To situate PLGA within the broader IA field, we briefly summarize selected non-PLGA sustained-release approaches—such as multivesicular liposomes, hyaluronic acid conjugates, and hybrid matrices—to contextualize comparative performance and safety. For proteins and peptides, central barriers include acidification inside degrading microspheres, aggregation during fabrication and storage, and incomplete or delayed release, as illustrated by glucagon-like peptide-1 analog formulations. Mitigation strategies span pH buffering, excipient-based stabilization, and gentler manufacturing that improve encapsulation efficiency and preserve bioactivity. Translation hinges on manufacturing scale-up and quality systems that maintain critical particle attributes and enable informative in vitro–in vivo interpretation. Clinically, prolonged symptom relief after single dosing has been demonstrated for corticosteroid depots (e.g., ~50% pain reduction over 12 weeks with a single PLGA–triamcinolone injection), whereas repeat-dose safety and indication expansion beyond the knee remain active needs best addressed through multicenter trials incorporating imaging and patient-reported outcomes. Consistent real-world performance will depend on controlling batch-to-batch variability and implementing pharmacovigilance approaches suited to long dosing intervals, enabling broader clinical adoption.

## 1. Introduction

Diseases affecting synovial joints, such as osteoarthritis (OA) and rheumatoid arthritis (RA), are among the leading causes of chronic pain and disability worldwide, with a global prevalence of 595 million people with osteoarthritis in 2020 (7.6% of global population) and 18 million people with rheumatoid arthritis worldwide and imposing substantial healthcare costs [1,2]. The economic burden is staggering, with osteoarthritis alone accounting for 1–2.5% of gross domestic product in developed countries, representing over USD 600 billion globally in annual healthcare expenditures and productivity losses [3], while significantly reducing quality of life through persistent joint pain, stiffness, and functional limitations.

Intra-articular (IA) injection represents a well-established therapeutic approach for joint disorders, offering the unique advantage of delivering drugs directly into the joint space to maximize local efficacy while minimizing systemic exposure [4]. This localized delivery strategy has become increasingly important as clinicians seek to optimize treatment outcomes while reducing the side effects associated with systemic medications. Common IA treatments include corticosteroids for short-term anti-inflammatory effects and pain relief, viscosupplementation with hyaluronic acid to restore joint lubrication, and more recently, platelet-rich plasma and stem cell therapies [5].

However, conventional IA therapies face a fundamental limitation: rapid clearance of injected agents from the joint space [6]. The synovial membrane consists of only a thin, discontinuous cellular layer with intercellular gaps ranging from 0.1 to 5 µm in size, providing minimal barrier function against drug egress [7]. This anatomical structure allows small molecules to diffuse rapidly into subsynovial capillaries and larger molecules or particles to be cleared primarily via lymphatic drainage from the joint capsule [8,9]. Consequently, most drugs injected into joints exhibit residence times of only hours to days, with therapeutic effects typically waning within 3–4 weeks [10].

This rapid clearance necessitates frequent repeat injections to maintain therapeutic benefit, introducing several clinical challenges including increased patient discomfort, healthcare costs, and risk of complications such as joint infection, bleeding, or inadvertent cartilage damage [11,12]. Furthermore, frequent corticosteroid injections have been associated with accelerated cartilage degradation in knee OA, raising concerns about long-term joint health [12,13]. The need for sustained-release IA drug delivery systems has become increasingly evident as the field seeks to prolong drug retention and therapeutic action within the joint space.

Poly(lactic-co-glycolic acid) (PLGA) has emerged as a highly promising biodegradable carrier platform to address these clinical needs [14]. As an FDA-approved biodegradable copolymer, PLGA undergoes predictable hydrolytic degradation in vivo, breaking down into its constituent monomers: lactic acid and glycolic acid [15,16]. These metabolites are naturally occurring compounds that are readily eliminated through normal physiological pathways, including the tricarboxylic acid cycle and renal excretion. This biocompatibility profile, combined with PLGA’s proven track record in other controlled-release pharmaceutical applications, has made it an attractive candidate for IA drug delivery. Given these advantages, PLGA-based systems represent a promising avenue for addressing the limitations of conventional IA therapies.

This review aims to provide an in-depth and practical analysis of PLGA-based IA drug delivery systems by detailing their fundamental polymer properties and degradation behaviors, summarizing current clinical applications including FDA-approved products and their limitations, exploring emerging biologic and non-PLGA platforms, and outlining critical translational considerations—regulatory pathways, manufacturing scale-up, and quality control challenges. The review concludes with evidence-based clinical implications, practical recommendations for formulation and administration, and near-term research and development prospects to expedite the integration of these innovative therapies into routine patient care.

## 2. PLGA Fundamentals for IA Delivery

### 2.1. Anatomical and Pharmacokinetic Considerations

Understanding the unique anatomical and physiological characteristics of synovial joints is crucial for designing effective PLGA-based delivery systems. The synovial joint represents a specialized anatomical compartment with distinct clearance mechanisms that significantly influence drug disposition and therapeutic outcomes. The synovial membrane forms the inner lining of the joint cavity and consists of two distinct layers: the intimal lining layer and the subintimal layer. The intimal layer, which is in direct contact with synovial fluid, is composed of specialized cells called synoviocytes arranged in a discontinuous monolayer, creating intercellular gaps of 0.1–5 µm that serve as the primary pathway for molecular exchange between the joint space and systemic circulation [17].

**Figure 1:**
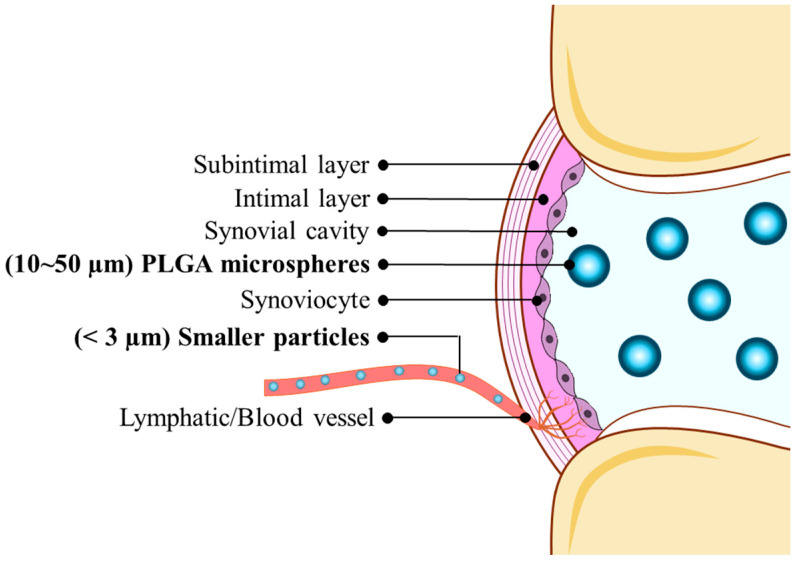
Particle Size-Dependent IA Retention and Clearance Mechanisms.

Synovial fluid itself is a specialized biological medium that differs significantly from plasma in composition and properties, containing high concentrations of hyaluronic acid which imparts viscoelastic properties essential for joint lubrication, as well as proteins, electrolytes, and various metabolites [12,18]. The volume of synovial fluid varies by joint size, ranging from approximately 0.5 mL in small joints to 3–4 mL in the knee joint under normal conditions, though this can increase significantly in diseased states [19]. Drug clearance from the joint occurs through multiple pathways that operate simultaneously, though clearance mechanisms vary considerably depending on particle size, surface properties, joint pathology, and individual patient factors, making precise size-based predictions challenging. While general trends suggest smaller particles (<300 nm) undergo more rapid clearance and larger particles (>10 μm) achieve better retention, significant variability exists across different drug formulations, disease states (healthy vs. inflamed joints), and patient populations [20,21]. The rate of clearance is influenced by molecular size, with smaller molecules being cleared more rapidly than larger ones, while for proteins and other macromolecules, lymphatic drainage becomes the predominant clearance mechanism. As illustrated in Figure 1, larger PLGA microspheres (~10–50 µm) are effectively retained within the joint cavity due to their inability to pass through small intercellular gaps (0.1–5 µm), whereas smaller nanoparticles (<1 µm) are rapidly cleared through lymphatic and systemic routes [20,21]. Given these complex and variable anatomical constraints, PLGA delivery system design must account for the dynamic nature of joint clearance mechanisms, with particle size optimization serving as a primary but not exclusive design parameter that must be balanced with polymer chemistry, drug properties, and patient-specific factors to achieve optimal therapeutic outcomes [19].

### 2.2. Polymer Chemistry and Degradation

PLGA is a synthetic aliphatic polyester formed by the copolymerization of two α-hydroxy acids: lactic acid (lactide) and glycolic acid (glycolide), with the polymer backbone consisting of ester linkages that are susceptible to hydrolytic cleavage under physiological conditions [16,22]. The degradation process follows a well-characterized bulk erosion mechanism where water penetrates throughout the polymer matrix, leading to random chain scission events, which ensures that drug release can occur throughout the polymer matrix as degradation proceeds rather than being limited to surface release [14,23,24].

Several key polymer parameters control degradation kinetics and drug release profiles as summarized in Table 1. Among the biodegradable polymers available for sustained-release IA applications, PLGA has emerged as the predominant choice for commercially approved injectable depot systems due to its optimal balance of degradation kinetics, biocompatibility, and regulatory precedent established through multiple FDA-approved products [14]. The lactide-to-glycolide ratio is perhaps the most important factor, with a higher lactide content resulting in more hydrophobic polymers that degrade more slowly [25]. For example, 75:25 lactide–glycolide PLGA typically degrades over 4–6 months, while 50:50 PLGA often shows the fastest degradation (2–3 months) due to optimal water uptake characteristics [25]. While other biodegradable polymers such as PLA and PCL are available, PLA’s slower degradation (>10 months) with higher hydrophobicity makes it less suitable for most IA therapeutic windows, and PCL’s extended degradation time (2–4 years) is more appropriate for long-term tissue engineering scaffolds rather than drug delivery applications [26,27]. Molecular weight significantly influences both mechanical properties and degradation time, with higher molecular weight PLGA polymers forming more robust particles and maintaining structural integrity longer [28]. End-group chemistry also plays a role, with acid-terminated PLGA undergoing autocatalytic degradation due to the catalytic effect of carboxylic acid end groups, while ester-capped PLGA shows more uniform degradation kinetics [29].

The glass transition temperature (T_g_) of PLGA varies with composition and molecular weight: 50:50 PLGA typically exhibits T_g_ of 45–50 °C, 75:25 PLGA shows T_g_ of 50–55 °C, and higher molecular weight formulations can reach T_g_ values of 55–60 °C, all ensuring the polymer remains in a glassy state at body temperature [30]. This property is critical for maintaining controlled release rather than immediate drug dump.

**Table 1:** Summary of PLGA characteristics influencing IA delivery.

Polymer Parameter	Options/Examples	Effect on Degradation & Release
Lactide:Glycolide Ratio	e.g., 50:50, 65:35, 75:25, 85:15	Higher lactide increases hydrophobicity and slows degradation; higher glycolide enhances hydration and accelerates release [29].
Molecular Weight (Mw)	Low (10–20 kDa), Medium (~50 kDa), High (>100 kDa)	Greater Mw provides stronger matrix integrity, leading to slower chain cleavage and prolonged release [31].
End-group Type	Acid-terminated vs. Ester-capped	Acid-terminated PLGA degrades faster via autocatalytic hydrolysis; ester-capped forms show more uniform erosion.
Morphology/Porosity	Solid non-porous microspheres vs. porous structures	Porous particles allow faster water penetration and a higher initial burst; dense microspheres sustain diffusion-controlled release.
Glass Transition (T_g_)	e.g., 45 °C vs. 55 °C (adjusted by copolymer ratio and Mw)	T_g_ above body temp means polymer remains glassy in situ. T_g_ slightly above 37 °C is ideal for mechanical stability of depot.
Formulation Additives	Stabilizers, plasticizers, pore-formers	Surfactants (e.g., PVA) or pore-formers increase initial burst, while plasticizers reduce Tg and accelerate diffusion.

### 2.3. Drug Release Mechanisms

IA drug release from PLGA-based depots typically proceeds in a multiphasic manner encompassing three distinct phases [14]. The initial burst phase occurs shortly after injection and results from the rapid diffusion of surface-localized or loosely entrapped drug [32]. Although the exact duration is formulation-dependent, this release generally occurs within the first hours to few days [33]. Reports suggest that a significant portion of the total drug load (typically ranging from 10 to 60%, though highly formulation-dependent) can be liberated in this phase, depending on factors such as particle size, drug loading, surface morphology, and processing method [34]. The magnitude of burst release varies considerably across different PLGA formulations and should be carefully controlled during development [33]. Burst release minimization techniques include surface smoothing via solvent annealing, porosity modulation through controlled drying conditions, and incorporation of release-retarding excipients such as albumin or magnesium stearate to form protective surface barriers [33].

Following this, a diffusion-governed sustained-release phase ensues. Water infiltrates the polymer matrix, initiating partial hydrolysis and forming aqueous channels through which drug molecules diffuse outward [35]. This stage supports prolonged delivery over several weeks to months [36]. Although some formulations exhibit near-linear release profiles, the rate typically decreases gradually due to increasing tortuosity and matrix compaction over time [37]. The structural evolution of PLGA carriers—swelling, pore formation, and onset of collapse—is discussed in detail in Section 2.4.

**Figure 2:**
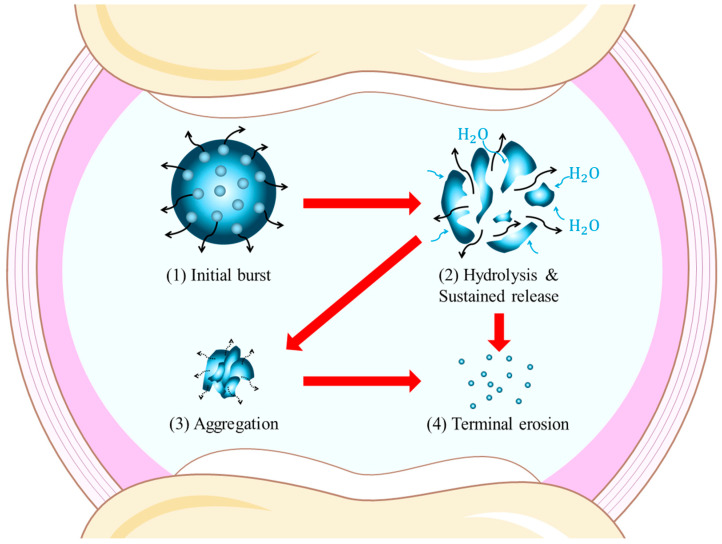
Drug release from PLGA microsphere in joint.

In the terminal erosion phase, progressive hydrolysis leads to fragmentation and solubilization of the polymer matrix, causing residual drug to be released [38]. In practice, complete erosion is generally expected; however, in intra-articular settings—given matrix dimensions/erosion mode (surface vs. bulk), limited vascularity and synovial sequestration, and potential microsphere aggregation under joint mechanics—PLGA particulates may transiently persist near the synovium and then continue hydrolyzing, ultimately proceeding to complete erosion [39]. These distinct drug release phases are schematically depicted in Figure 2, clearly demonstrating the initial burst, sustained diffusion-controlled release, and terminal erosion phases associated with PLGA-based depots in the IA environment.

### 2.4. Size-Dependent Behavior: Microspheres Versus Nanoparticles

The biological fate and therapeutic efficacy of PLGA-based delivery systems in IA applications are fundamentally governed by particle size, which determines clearance pathways, tissue penetration, and residence time within the joint space [21].

#### 2.4.1. Clearance Pathways by Size

Particle clearance from the joint space follows distinct, size-dependent pathways determined by the anatomical characteristics of synovial joints. The synovial membrane consists of a discontinuous cellular layer with intercellular gaps ranging from 0.1 to 5 μm, serving as the primary determinant of particle retention versus clearance [21]—and this layer becomes more highly vascularized and permeable under inflammatory conditions [40].

Small nanoparticles (<300 nm) are generally cleared more rapidly from both healthy and inflamed joints, primarily through passive diffusion across the synovial membrane, with residence times typically ranging from hours to days. Notably, synovial inflammation markedly accelerates this clearance by increasing capillary perfusion and lymphatic drainage: inflamed arthritic synovium undergoes angiogenesis and lymphangiogenesis that facilitate nanoparticle egress [40,41]. For instance, rheumatoid joints exhibit increased vascular fenestrations and lymph flow, leading to faster NP clearance compared to healthy joints [41]. Pathological joint lesions can also influence distribution and retention; osteoarthritic cartilage degeneration, for example, raises cartilage matrix porosity and fluid flow [42], which may enable deeper nanoparticle penetration into cartilage fissures (altering their retention location). Medium-sized particles (300 nm to 3 μm) exhibit intermediate clearance behavior, primarily via lymphatic drainage, and inflammation-enhanced lymph flow can hasten their removal from the joint space [41]. Larger microspheres (>10 μm) tend to be retained in the joint through size-exclusion, though complete retention is not absolute—severe inflammation can increase synovial membrane permeability enough that even some microparticles in the low micrometer range may escape into circulation, whereas such particles would be immobilized in a normal joint [40].

#### 2.4.2. Tissue Penetration Barriers

The dense extracellular matrix of cartilage presents additional size-dependent barriers. In cartilage tissue, collagen fibrils range from approximately 30–80 nm in diameter with interfibrillar spacing of ~100 nm [43], while the dense proteoglycan networks (primarily aggrecan aggregates) create nanoscale interfibrillar spaces that restrict diffusion of larger molecules [44,45].

The hierarchical organization of this collagen–proteoglycan matrix creates size-selective barriers, with effective pore dimensions varying significantly depending on tissue depth and pathological state [46]. Nanoparticles smaller than 15 nm can penetrate the full thickness of healthy cartilage, particles in the 15–60 nm range can penetrate superficial cartilage layers, while larger particles (>60 nm) are generally restricted to the cartilage surface [47,48].

#### 2.4.3. Surface Charge Effects

Surface charge represents a critical parameter that can override size-based predictions. Cationic PLGA nanoparticles can form micrometer-sized aggregates upon contact with anionic hyaluronic acid in synovial fluid through electrostatic cross-linking, effectively converting nanoparticles into larger retention-promoting structures [49]. This size amplification strategy has demonstrated remarkable efficacy, with cationic nanoparticles showing over 50% retention at 28 days compared to rapid clearance of free drug within 3 days [49].

The electrostatic interactions between charged particles and synovial fluid components can be strategically exploited to modulate both retention and distribution patterns [19]. However, excessive positive charge may also trigger inflammatory responses or alter normal joint physiology, requiring careful optimization of surface properties [19].

#### 2.4.4. Selection Criteria for Therapeutic Applications

The choice between microspheres and nanoparticles should be guided by specific therapeutic objectives and target sites within the joint.

**Microsphere systems (20–50 μm)** are optimal for sustained drug delivery applications where prolonged joint residence is prioritized, as exemplified by Zilretta^®^ [50]. These larger particles:

- Provide extended depot effect through size exclusion.
- Minimize systemic exposure through reduced clearance.
- Are suitable for anti-inflammatory drugs requiring sustained local concentrations.

**Nanoparticle systems (25–40 nm)** are preferable when tissue penetration is required, offering the best compromise between penetration capability and retention time [51]. These smaller particles:

- Can access cartilage matrix for chondrocyte-targeted delivery.
- Enable deeper tissue penetration for regenerative applications.
- Allow for surface modifications to enhance cellular uptake.

Hybrid systems such as nanoparticles-in-microspheres combine the prolonged retention of larger microspheres with the deep tissue penetration capabilities of smaller nanoparticles, potentially offering the benefits of both approaches while mitigating individual limitations [52].

The selection process must also consider drug properties, dosing requirements, and safety profiles, with particle size optimization representing a critical design parameter that directly impacts therapeutic outcomes in IA applications. However, practical considerations such as injection needle requirements (larger bore needles for microspheres vs. standard needles for nanoparticles) and manufacturing complexity must be balanced against therapeutic benefits, as demonstrated by the clinical success of Zilretta^®^’s microsphere approach despite requiring specialized injection protocols [53].

### 2.5. Biocompatibility and Sterilization Considerations

Biocompatibility is a critical prerequisite for any IA formulation, particularly due to the sensitive, enclosed nature of the synovial joint. PLGA polymers exhibit excellent safety profiles, with their lactic and glycolic acid degradation byproducts efficiently metabolized via natural pathways [14,16]. Preclinical and clinical studies have confirmed that well-designed PLGA microspheres elicit only mild, transient inflammation that subsides with degradation [54]. The magnitude of local immune response is influenced by particle size, surface chemistry, polymer purity and characteristics of active pharmaceutical ingredients.

Sterility remains essential to mitigate the risk of septic arthritis. Gamma irradiation is commonly employed and typically maintains PLGA polymer integrity [55]. For formulations containing labile biologics, aseptic manufacturing and filtration-based approaches are preferred. Quality control strategies encompass drug loading, particle size uniformity, endotoxin levels, and release kinetics.

From a formulation design perspective, several strategies enhance local tolerability and performance. Incorporating buffering agents such as magnesium hydroxide or MgCO_3_ can neutralize acidic microenvironments formed during PLGA degradation, reducing the risk of pH-triggered inflammation [56]. Surface PEGylation or inclusion of anti-fouling polymers may reduce protein adsorption and modulate immune cell activation [14,57]. Additionally, controlling initial burst release through surface smoothing or porosity modulation can minimize joint irritation and extend therapeutic coverage [58].

## 3. Current Clinical Applications

### 3.1. Extended-Release Corticosteroids: Zilretta^®^ as the Pioneer

The FDA approval of Zilretta^®^ (extended-release triamcinolone acetonide) in October 2017 marked a watershed moment in osteoarthritis therapy, becoming the first extended-release IA corticosteroid for knee OA pain (Table 2 [50]). Zilretta^®^ consists of 32 mg triamcinolone acetonide in biodegradable 75:25 PLGA microspheres (~45 µm) that release drug over ~12 weeks [50]. In pivotal trials, one injection achieved ~50% pain reduction for up to 3 months—versus 6–8 weeks with immediate-release steroids—and produced lower peak plasma steroid levels, indicating reduced systemic exposure.

Despite these advantages, real-world use remains confined to single injections pending repeat-dosing safety data [50]. A Phase IIIb open-label study showed repeat injections at ~3 months provided comparable pain relief but increased mild-to-moderate injection-site arthralgia from 6% after the first dose to 16% after the second; no serious adverse events or cartilage damage were observed. Additional limitations include knee-only indication and exclusion of patients with uncontrolled diabetes or significant comorbidities, and higher cost requiring pharmacoeconomic justification. Until further repeat-dose safety and cost-effectiveness data emerge, Zilretta^®^ is generally reserved for one-time or infrequent use in chronic knee OA.

### 3.2. Advanced PLGA Corticosteroid Formulations

Building on the success of Zilretta, novel PLGA-based steroid depots are being developed to further extend duration of action and improve safety. A leading example is EP-104IAR, an investigational extended-release fluticasone formulation for knee OA [59]. EP-104IAR uses a proprietary “Diffusphere” microsphere technology to encapsulate fluticasone propionate crystals in a biodegradable polymer shell, enabling extremely prolonged drug release (potentially months to a year). In the Phase II SPRINGBOARD trial, a single 25 mg injection of EP-104IAR produced clinically meaningful pain relief for up to ~14 weeks, significantly longer than placebo-treated controls [59]. By week 12, WOMAC pain scores had improved significantly more with EP-104IAR than with vehicle (mean change −2.89 vs. −2.23, *p* ≈ 0.004) [59], and a significant benefit persisted through week 14 [59].

Importantly, systemic exposure to fluticasone was minimal—the formulation exhibited a blunted initial plasma peak and an estimated IA half-life of ~18–20 weeks [59]. These results suggest EP-104IAR can safely provide longer-lasting steroid therapy than current IA injections (Zilretta or immediate-release steroids) [60]. Phase III studies (including evaluation of repeat and bilateral dosing) are in development to confirm its efficacy and safety [59]. If successful, EP-104IAR could represent a biannual or annual injectable treatment for osteoarthritis pain, significantly reducing injection frequency compared to existing options.

### 3.3. NSAIDs and Anti-Inflammatory Drug Delivery Systems

Nonsteroidal anti-inflammatory drugs (NSAIDs) are another class of therapeutics under investigation for IA sustained release, given their ability to reduce pain and inflammation if delivered locally without systemic side effects [61]. To date, no PLGA-based NSAID depot has reached human clinical trials, but preclinical studies are encouraging. For example, celecoxib, a selective COX-2 inhibitor, has been encapsulated in PLGA microspheres in animal models, achieving sustained drug release for approximately 6–10 weeks [62]. Such a formulation could provide extended joint relief while avoiding the gastrointestinal and cardiovascular risks of systemic NSAIDs. Early studies also suggest that targeting COX-2 within the joint (while sparing COX-1 activity elsewhere) may be beneficial, as COX-2 is upregulated in inflamed arthritic joints [63]. Other NSAIDs like indomethacin and diclofenac have similarly been formulated in experimental PLGA nanoparticle or microparticle systems, showing prolonged anti-inflammatory effects in arthritic animal joints [64].

While these PLGA–NSAID approaches remain in the preclinical stage, non-PLGA sustained-release NSAID formulations have demonstrated clinical success, particularly the HA-conjugated diclofenac system approved in Japan (SI-613), which validates the therapeutic potential of locally delivered NSAIDs for joint disorders [65]. We can expect that in the coming years, improved polymer formulations or conjugates will bring NSAID depots into clinical trials, expanding the options for long-acting joint analgesia.

### 3.4. Disease-Modifying and Regenerative Approaches

Beyond symptomatic relief, researchers are actively investigating PLGA-based delivery systems for disease-modifying antirheumatic drugs (DMARDs) and regenerative therapeutics. The goal is to locally administer agents that could alter the course of joint disease (e.g., rheumatoid arthritis or cartilage degeneration) rather than only treat pain. One example is IA methotrexate (MTX) loaded in PLGA microparticles, aimed at treating inflammatory arthritis in affected joints [66]. Preclinical studies of MTX–PLGA formulations have shown prolonged drug retention in the joint and lower systemic exposure compared to systemic MTX, suggesting the potential for enhanced efficacy with reduced side effects [66]. Advanced designs are even incorporating targeting moieties and triggerable release mechanisms—for instance, PLGA microspheres embedded with gold nanoparticles to enable near-infrared triggered MTX release [67]—which have yielded promising results in animal models.

In the context of osteoarthritis, regenerative medicine strategies are also being combined with sustained-release delivery [68]. An intriguing approach has been the use of low-dose dexamethasone encapsulated in PLGA microspheres to modulate the joint environment and promote cartilage health [69]. Rather than a one-time high dose for pain relief, these formulations slowly release dexamethasone over many weeks, aiming to reduce inflammatory catabolism and aid cartilage matrix maintenance [69]. In preclinical studies, dexamethasone-loaded PLGA microspheres provided drug release for up to 99 days and demonstrated chondroprotective effects in vitro and in animal models [69]. This suggests that sustained delivery of certain anti-catabolic or anabolic factors could potentially slow cartilage degeneration. Similarly, research is underway on PLGA delivery of growth factors (e.g., BMP-7, IGF-1) or cytokine inhibitors (e.g., IL-1 receptor antagonist) to spur cartilage repair or quell synovial inflammation for extended periods [70].

At present, none of these PLGA-based DMARD or regenerative therapies have entered human trials, but they represent the next frontier in IA treatment. By concentrating potent disease-modifying agents in the joint over long durations, these approaches aspire to not only relieve symptoms but also alter the trajectory of joint diseases like OA and RA. The coming years will reveal whether such PLGA-based formulations can safely translate to clinical use and deliver true disease-modifying benefits.

### 3.5. Alternative Non-PLGA Sustained-Release Platforms

To provide broader context for PLGA approaches, Table 3 summarizes key non-PLGA intra-articular sustained-release products that have reached clinical evaluation. These examples illustrate complementary delivery strategies and clinical performance benchmarks.

#### 3.5.1. TLC599: Multivesicular Liposome Technology

TLC599 utilizes multivesicular liposome technology, specifically DepoFoam^®^, to achieve sustained release of dexamethasone sodium phosphate [20,71]. The system consists of non-concentric lipid bilayers creating multiple internal aqueous compartments separated by phospholipid membranes [71]. In comparison to conventional PLGA system, the liposomal DepoFoam^®^ platform also achieves efficient encapsulation of a hydrophilic drug, which is challenging for hydrophobic PLGA matrices (PLGA often shows low encapsulation efficiency and high burst for water-soluble drugs) [72,73]. The release mechanism involves a biphasic pattern: initial rapid release from surface-accessible chambers, followed by sustained release from internal vesicles through membrane erosion and coalescence processes [74]. This architecture enables drug retention in synovial fluid for up to 120 days, providing extended pharmacokinetic benefits compared to conventional corticosteroid formulations [75]. By avoiding polymer degradation, TLC599 produces no acidic byproducts, potentially improving intra-articular biocompatibility (no pH drop and less irritation) [71]. On the other hand, these advantages come with increased manufacturing complexity: the multivesicular liposome process is specialized and may raise production costs compared to standard PLGA microsphere fabrication [76].

#### 3.5.2. Cingal^®^: Triamcinolone-Modified Hyaluronic Acid Composite

Cingal^®^ represents a physical mixture combining cross-linked sodium hyaluronate (88 mg) with triamcinolone hexacetonide (18 mg) [77]. Unlike PLGA systems that require complex drug encapsulation within polymer matrices, Cingal employs a physical mixture approach where the steroid is cross-linked with hyaluronic acid, eliminating encapsulation efficiency concerns and ensuring consistent drug loading [77]. This cross-linking strategy simplifies manufacturing compared to PLGA microsphere fabrication, as it involves established cross-linked HA production processes without requiring complex multi-step encapsulation procedures [78]. The corticosteroid provides immediate anti-inflammatory effects through prostaglandin E2 synthesis inhibition [79], while the cross-linked hyaluronic acid matrix functions as a viscoelastic depot [80]. The cross-linking of hyaluronic acid, typically achieved through 1,4-butanediol diglycidyl ether chemistry, creates a three-dimensional network that extends drug residence time and provides mechanical joint support [81]. The dual-action mechanism enables rapid symptom relief within 1–3 weeks followed by sustained benefits through 26 weeks [77]. The use of hyaluronic acid, a naturally occurring biopolymer in synovial fluid, provides inherent biocompatibility advantages over synthetic PLGA polymers, resulting in excellent tolerability with no serious adverse events in clinical trials and only transient mild side effects typical of viscosupplements [82].

#### 3.5.3. SI-613 (Joyclu^®^): Drug-Polymer Conjugate Technology

SI-613 employs covalent conjugation of diclofenac to hyaluronic acid via 2-aminoethanol linkers attached to glucuronic acid moieties [83]. This drug-polymer conjugate creates an inactive prodrug that undergoes enzymatic hydrolysis within the synovial environment to release active diclofenac in a controlled manner [83,84]. Unlike PLGA microsphere systems, which physically encapsulate drug within a biodegradable synthetic polymer matrix and often face challenges with encapsulation efficiency and initial burst release [72], SI-613 chemically binds the drug to a naturally derived HA backbone, ensuring all diclofenac is stably incorporated and released only via enzymatic cleavage. The conjugation fundamentally alters the biological activity: SI-613 uniquely stimulates the production of high molecular weight hyaluronic acid (>2400 kDa) through enhanced hyaluronan synthase 2 (HAS2) expression and suppressed hyaluronidase 2 (HYAL2) expression [85]. This covalent modification enables specific molecular recognition pathways that promote endogenous HA synthesis and matrix homeostasis, a property not observed with PLGA carriers, which lack intrinsic biological activity and do not modulate joint matrix metabolism. This mechanism differs from simple physical mixtures, as the covalent modification enables specific molecular recognition pathways that promote endogenous HA synthesis and matrix homeostasis [86]. From a process-development perspective, SI-613 is produced via defined chemical conjugation and downstream purification to control the drug-to-polymer substitution ratio, whereas PLGA microspheres are typically manufactured by multi-step emulsification/solvent removal and drying, processes that inherently introduce variability in drug loading and early-phase release kinetics due to emulsion stability, solvent removal rate, and porosity formation.

#### 3.5.4. Clinical Implications and Regulatory Status

While PLGA microspheres (exemplified by Zilretta^®^) pioneered extended-release intra-articular therapy, these platforms (PLGA and non-PLGA) each offer distinct performance profiles suited to different clinical scenarios. TLC599 employs physical encapsulation within lipid compartments, Cingal^®^ utilizes matrix-based co-delivery, and SI-613 leverages chemical conjugation for prodrug activation. The multivesicular liposome technology provides the longest depot effect (24+ weeks), while the conjugate system uniquely modifies tissue biology through enhanced endogenous HA production. Cross-linked HA matrices offer intermediate duration with rapid onset, balancing immediate and sustained therapeutic effects [87]. In practice, platform selection should align with target release duration, onset needs, and comorbidity profile, recognizing that no single system is universally superior. Each approach addresses limitations of conventional IA therapies: rapid synovial clearance, limited duration of action, and potential chondrotoxicity. The sustained-release mechanisms enable reduced injection frequency while maintaining therapeutic drug concentrations within the joint space, potentially improving patient compliance and clinical outcomes in osteoarthritis management [88]. For prolonged steroid effect with fewer visits, TLC599 offers ~6-month coverage; for single-dose, well-characterized 3–4 month relief with lower systemic exposure, PLGA (Zilretta^®^) remains a benchmark; for rapid symptom relief with viscoelastic support, Cingal^®^ provides immediate onset plus HA benefits; and for steroid-sparing needs, SI-613 enables NSAID-based control with attention to rare hypersensitivity risk. Accordingly, choice should balance duration and onset with biocompatibility and practical manufacturing factors, as summarized in the horizontal comparison above.

## 4. Emerging Biologic Therapeutics

Localized delivery of biologic agents via PLGA depots holds the promise of alleviating joint symptoms and potentially modifying disease progression by sustaining therapeutic concentrations within the synovial space. However, clinical translation of PLGA-based biologics faces major hurdles: regulatory uncertainty over combination-product classification, safety concerns including immunogenicity and off-target effects, manufacturing challenges in achieving consistent biologic loading and controlled release and limited long-term safety data for novel RNA and gene therapy modalities. PLGA-based IA systems can concentrate drugs at the site of pathology, reduce systemic exposure, and maintain release over weeks to months, thereby improving safety and efficacy profiles. Table 4 summarizes representative PLGA-based biologic delivery platforms, and the following subsections discuss each modality in greater detail.

### 4.1. TNF-α Inhibitors and Advanced Immunomodulators

TNF-α inhibitors are among the most extensively studied biologics for PLGA encapsulation, with infliximab, adalimumab, and etanercept successfully loaded into PLGA microspheres [89]. Research by Lamela-Gómez et al. demonstrated improved encapsulation of infliximab in PLGA microspheres using ultrasonic atomization technique, achieving encapsulation efficiencies of 70–80% compared to conventional methods (17–23%) while maintaining antibody bioactivity [90]. The resulting microspheres provided sustained infliximab release for approximately 3 weeks in vitro, with released antibody retaining full TNF-α neutralization activity and demonstrating significant anti-inflammatory effects in cellular models [90].

siRNA-based TNF-α inhibition has also shown promise: Presúmey et al. developed PLGA microspheres encapsulating anti-TNF-α siRNA, with sustained release over several weeks and high encapsulation efficiency [98]. In a murine arthritis model, IA administration inhibited TNF-α expression for >14 days, illustrating the potential of RNA interference-based anti-rheumatic therapy [91].

### 4.2. Interleukin-1 (IL-1) Pathway Modulation

IL-1 receptor antagonist (IL-1Ra, anakinra) is a natural inhibitor of IL-1 signaling with proven efficacy in rheumatoid arthritis and osteoarthritis [92]. In a Phase I trial (NCT00110916) involving 170 OA patients, a single high-dose IA injection showed good tolerability but limited long-term benefits; pain reduction lasted 4 days with no significant differences vs. placebo at 4 weeks, and serum half-life was ~4 h [93,94].

PLGA microspheres loaded with IL-1Ra demonstrated 4–6 weeks of sustained release in vitro and potent anti-inflammatory effects in multiple preclinical models [92,99]. Gorth et al. showed that PLGA 50:50 microspheres attenuated IL-1β–induced degeneration in nucleus pulposus constructs for up to 20 days, with complete inhibition of inflammatory mediators for the first week [92]. Magnetic-targeted PLGA microspheres have been developed to further enhance delivery specificity [90].

### 4.3. Growth Factors and Regenerative Medicine Applications

Growth factors represent a promising approach for PLGA-based intra-articular delivery, potentially promoting cartilage repair rather than merely managing symptoms [84,90]. Three representative strategies are outlined at the outset: TGF-β1 for promoting chondrogenesis, dual delivery of bFGF and IGF-1 for synergistic cartilage regeneration, and bone morphogenetic proteins (BMPs) for osteochondral repair.

TGF-β1 has been extensively studied for cartilage regeneration, enhancing chondrogenesis via Smad and MAPK signaling [95]. PLGA microspheres loaded with TGF-β1 release over 4–8 weeks and stimulate cartilage matrix synthesis in vitro and in vivo [95,96]. Dual bFGF/IGF-1 delivery systems show synergistic cartilage regeneration when co-released from PLGA carriers [95,100]. BMPs have been encapsulated with protective carriers to preserve bioactivity during processing, supporting both bone and cartilage formation for osteochondral defect repair [101].

Challenges include maintaining bioactivity during encapsulation and sustained release, given growth factors’ sensitivity to acidic microenvironments, oxidative stress, and mechanical shear [93]. Advanced stabilization strategies such as co-encapsulation of protective excipients and pH buffers have been developed to address these limitations (Table 5).

### 4.4. Gene Therapy and Nucleic Acid Delivery

PLGA-based gene therapy offers sustained production of therapeutic proteins within joint tissues [97,98]. PLGA nanoparticles (150–400 nm) carrying IL-1Ra plasmid DNA have produced weeks-to-months of gene expression and anti-inflammatory effects in arthritic animal models [97]. p66shc siRNA–loaded PLGA nanoparticles (~180 nm) provided 48 h of sustained release and maintained gene silencing for 21 days in MIA-induced OA rats, reducing cartilage damage and pain behaviors [97].

Delivery efficiency improves with surface modifications, including cationic polymers for electrostatic interaction, cell-penetrating peptides for intracellular trafficking, and ligand-mediated targeting (e.g., hyaluronic acid for CD44 on chondrocytes) [106]. However, achieving efficient intracellular trafficking, stability within the acidic microenvironment, and precise cell-specific targeting remain critical challenges for clinical translation.

### 4.5. Formulation Challenges & Advanced Stabilization Strategies

The successful development of PLGA-based delivery systems for biologics requires addressing formulation challenges that are fundamentally different from those encountered with small molecules. Protein therapeutics are particularly vulnerable due to their complex tertiary structures and sensitivity to manufacturing, storage, and release conditions. During encapsulation into PLGA microspheres—often via double emulsion processes—proteins are exposed to organic solvents, mechanical shear, and interfacial stresses [107,108]. These stresses can trigger unfolding, aggregation, and loss of activity, representing critical barriers to maintaining therapeutic efficacy.

An additional complication arises from the acidic microenvironment generated during PLGA hydrolysis. As lactic and glycolic acid degradation products accumulate, pH within the microsphere interior can drop to 1.5–3.5 [109] conditions that may persist for weeks [110]. Such prolonged acidity can irreversibly damage protein structure, compromise bioactivity, and destabilize release kinetics. These challenges create a cascade of interrelated problems: acidic degradation conditions lead to protein instability, which in turn causes aggregation and surface adsorption, ultimately resulting in incomplete drug release from the polymer matrix [109].

#### 4.5.1. Clinically Relevant Example: GLP-1 Microsphere Challenge

To illustrate the specific manifestation of these challenges, recent investigations into GLP-1 receptor agonists demonstrate how these acidic microenvironment issues directly impact peptide systems. Formulating GLP-1 analogs such as semaglutide and liraglutide in PLGA microspheres has frequently resulted in incomplete release profiles and suboptimal bioavailability [111,112]. The mechanism underlying this incomplete release is directly linked to the acidic microenvironment: as PLGA degrades, the internal pH drops to 2–3, causing GLP-1 analogs to undergo acid-catalyzed degradation pathways including deamidation of asparagine residues and peptide bond hydrolysis [113]. For example, semaglutide-loaded systems often display a substantial “free drug” fraction that is rapidly released, followed by incomplete liberation of encapsulated peptide [112,114]. The acidic conditions within degrading microspheres cause semaglutide to form acid-labile covalent bonds with PLGA degradation products, effectively trapping approximately 50% of the payload within the polymer matrix even after complete erosion [114]. Structural differences between semaglutide’s C18 fatty acid linker (with two carboxylic acid groups) and liraglutide’s C12 linker (single carboxylic acid) alter drug–polymer interactions, leading to variable release kinetics; in some cases, only ~50% of the payload is released over extended periods, with the remainder trapped in the polymer matrix [114,115]. This acid-mediated drug entrapment represents a direct consequence of uncontrolled pH conditions within PLGA microspheres. These case studies underscore the need for targeted stabilization and release-modulation strategies tailored to the physicochemical profile of each biologic.

#### 4.5.2. Advanced Stabilization Strategies

To address these multifaceted challenges, several advanced stabilization strategies have been developed and validated (Table 5). pH buffering systems incorporating basic salts such as magnesium hydroxide (Mg(OH)_2_) or magnesium carbonate (MgCO_3_) directly counteract the acidic microenvironment by neutralizing lactic and glycolic acid degradation products, maintaining physiological pH conditions (6.5–7.4) throughout the release process [56,102]. These buffering approaches have demonstrated significant improvements in protein stability and release kinetics. Specifically for GLP-1 systems, co-encapsulation of Mg(OH)_2_ has been shown to increase complete drug release from 45–50% to over 85% by preventing acid-catalyzed aggregation and covalent modification of the peptide [56,102].

Disaccharide stabilizers, particularly trehalose, function as molecular chaperones to preserve protein tertiary structure during both processing and long-term storage [103]. Advanced processing techniques, including ultrasonic atomization, have demonstrated 3–4-fold improvements in encapsulation efficiency compared to conventional methods while minimizing protein exposure to denaturing conditions [104]. Ultrasonic atomization achieved encapsulation efficiencies of 70–80% for complex proteins compared to only 17–23% with conventional emulsion/evaporation methods [105]. This improvement directly addresses incomplete release issues by ensuring more uniform drug distribution within microspheres, reducing surface localization that contributes to burst release and subsequent aggregation.

#### 4.5.3. Incomplete Drug Release: A Persistent Challenge

Incomplete payload release from PLGA-based biologic depots remains a significant barrier to consistent therapeutic outcomes [116]. The main cause of this incomplete release lies in the acidic microenvironment created during PLGA degradation: proteins trapped within microspheres experience pH conditions as low as 1.5–2.5, leading to irreversible structural changes, aggregation, and covalent modification that prevent normal diffusion-based release [117,118]. Even after polymer erosion, up to half of the encapsulated protein or peptide can remain trapped within the matrix, resulting in subtherapeutic dosing and variable efficacy [119]. The initial burst release may flood the joint with high local concentrations, risking off-target effects, while the residual fraction limits long-term delivery. Extended exposure to the acidic microenvironment formed by PLGA degradation can further denature proteins, promote aggregation, and increase immunogenicity [120]. Addressing these issues requires precise tuning of polymer composition, particle porosity, and excipient selection to balance burst and sustained phases, neutralize internal acidity, and achieve predictable, complete release of biologic agents.

## 5. Clinical Translation and Regulatory Considerations

### 5.1. Regulatory Framework and Combination Product Classification

PLGA-based IA drug delivery systems are classified as combination products under 21 CFR 3.2(e), where the drug provides the primary mode of action and the polymer controls release through physical mechanisms [121]. Most fall under the review body responsible for drugs; however, biologic-led IA depots, such as those containing IL-1 receptor antagonist, may be reviewed by the authority overseeing biologics [122]. Table 6 provides a comprehensive overview of the regulatory and CMC workflow stages for PLGA-based IA depot development, from initial classification through post-marketing surveillance.

The Zilretta^®^ case (NDA 208845) established a critical precedent. At the time of its NDA review, the use of PLGA in an approved IA drug was unprecedented [123,124], leading to the requirement for route-specific toxicology, local tolerability assessment, and evaluation of pharmacokinetic modifications associated with sustained release. The Pre-Request for Designation (Pre-RFD) process allows sponsors to obtain early guidance on classification and development expectations [125]. In practice, sponsors typically engage early with FDA’s Office of Combination Products (OCP) via Pre-RFD to confirm product classification and lead center, then proceed to a pre-IND meeting to align on IA route–specific nonclinical plans (e.g., local tolerance in a weight-bearing joint), CMC expectations for PLGA microspheres (critical attributes and release testing), and clinical endpoints appropriate for multi-month depots [121,125].

### 5.2. Chemistry, Manufacturing, and Controls (CMC) Requirements

CMC evaluation must address both the active pharmaceutical ingredient and the PLGA matrix, including manufacturing/storage interactions and their effect on performance. Critical Quality Attributes (CQAs) include particle size and morphology, drug loading, in vitro release, residual solvents, polymer molecular weight, and lactide–glycolide ratio, as well as sterility, endotoxins, and stability [126].

Characterization typically employs multiple orthogonal methods, while dissolution testing for IA microspheres may use dialysis, sample-and-separate, or flow-through cell (USP apparatus 4) methods to better simulate the joint environment [127]. Establishing an in vitro–in vivo correlation (IVIVC) for intra-articular formulations is challenging due to patient-specific variations in joint movement, synovial fluid composition, and clearance pathways, which complicate prediction of in vivo behavior from in vitro release data [128].

Quality by Design principles link Critical Process Parameters—such as emulsification conditions, phase ratios, temperature control, solvent removal kinetics, and drying conditions—to final CQAs [129]. Process Analytical Technology (PAT) tools, as outlined in regulatory guidance [130], include Focused Beam Reflectance Measurement for real-time particle sizing and near-infrared spectroscopy for solvent and moisture monitoring, enabling enhanced process control. As a real-world example, Zilretta^®^’s NDA (208845) documents a QbD-aligned approach in which defined CPPs (e.g., emulsification and solvent-removal conditions) were linked to CQAs (microsphere size distribution, drug loading, and in vitro release), and clinical–commercial comparability was established with late-phase lots manufactured under the intended commercial process; FDA’s multidisciplinary reviews discuss IA route-specific toxicology and the sustained-release PK considerations required for this PLGA microsphere product [123,124,126].

### 5.3. Clinical Trial Design and Regulatory Considerations

Sustained-release intra-articular formulations require clinical trial designs that capture both onset and durability of therapeutic effect, necessitating follow-up periods well beyond those typical of conventional corticosteroid trials [131]. Primary efficacy endpoints should be timed to coincide with the expected peak benefit while also documenting the duration of meaningful effect, using validated pain and function measures coupled with supportive secondary assessments such as quantitative imaging or biochemical biomarkers of joint metabolism [132,133].

Eligibility criteria must account for the unique risk profile of depot products, including potential cumulative exposure from repeat dosing [134]. Safety monitoring plans should incorporate targeted joint imaging and, where feasible, minimally invasive sampling of synovial fluid to detect early changes in joint health [135]. The operational aspects of sustained-release IA trials—such as centralized capture of patient-reported outcomes, standardized functional testing, and consistent imaging protocols across study sites—are critical to generating interpretable long-term data [136].

Experience with repeat dosing of Zilretta^®^ has demonstrated the maintenance of efficacy up to a second injection but has also underscored the need for additional safety data on cumulative joint exposure [137]. Comparable longitudinal datasets will be required for emerging PLGA-based depots and other novel intra-articular therapeutics to establish both their sustained benefit and long-term safety profile [133].

### 5.4. Safety Evaluation and Risk Assessment

Safety assessment combines established polymer biocompatibility data with IA-specific toxicology, accounting for the confined joint space, limited clearance pathways, and mechanical loading [123]. Zilretta^®^ safety studies identified mild local tissue changes and dose-related cartilage findings, underscoring the need for histological evaluation of target tissues in relevant animal models [124].

For biologic-loaded PLGA depots, the acidic microenvironment inside degrading microspheres can reach pH 2–3, promoting protein aggregation and potential immunogenicity; mitigation strategies include buffering agents, protective excipients, and controlled-release design. Risk–benefit assessment must consider possible persistent or cumulative effects from depot exposure alongside the clinical advantages of reduced injection frequency.

Long-term monitoring should use advanced imaging modalities such as quantitative MRI and ultrasound, combined with robust post-marketing surveillance to detect delayed adverse events [138,139]. Favorable risk–benefit profiles, as demonstrated by Zilretta^®^, can be achieved when development incorporates comprehensive safety evaluation and adherence to evidence-based dosing regimens [50].

## 6. Manufacturing Scale-Up and Quality Systems

### 6.1. Commercial Manufacturing Technologies and Scale-Up Challenges

**Figure 3:**
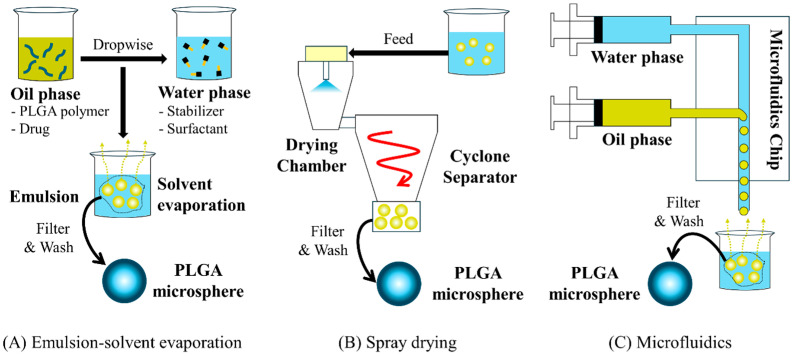
Comparison of manufacturing methods for commercially available PLGA products.

Transitioning from laboratory-scale to commercial production of PLGA-based intra-articular (IA) products requires control of scale-dependent parameters such as emulsification energy, phase ratios, temperature, and solvent removal kinetics [129,139]. The main commercial manufacturing routes—emulsion–solvent evaporation, spray drying, and microfluidics—differ in scalability, particle attributes, and process control needs (Figure 3). Emulsion–solvent evaporation remains the most widely adopted approach, as seen in Zilretta^®^, where commercial-scale processes were established during Phase II to ensure Phase III material reflected final manufacturing conditions [140]. In parallel, spray drying enables continuous processing and may suit thermally stable drugs, although it commonly yields smaller particles and demands careful thermal management [141,142]. Microfluidics offers narrow size distribution and precise control over particle formation; however, throughput limitations necessitate numbering-up or continuous-flow strategies. Recent work has demonstrated scale-up while maintaining tight control of particle characteristics [142,143]. Accordingly, while some contract manufacturing organizations have invested in PLGA capabilities, such expertise remains relatively limited [144], and technology choice should balance feasibility, cost, regulatory expectations, and supply chain integration.

### 6.2. PAT and Quality Control Systems

Consistent with fit-for-purpose CMC evaluation, PAT supports real-time process monitoring and quality control for PLGA microsphere production [145,146]. Tools such as Focused Beam Reflectance Measurement (FBRM) can track particle size during emulsification, though accuracy may be reduced for transparent or semi-transparent particles [146,147].

In vitro release testing for injectable microspheres is technically demanding; established approaches include dialysis, sample-and-separate, and flow-through cell (USP 4) methods [148]. Analytical characterization employs laser diffraction, microscopy, DSC, and, for biologics, methods such as circular dichroism spectroscopy and bioactivity assays [130,149]. Stability testing must assess both chemical and physical integrity, with accelerated conditions designed to avoid artifacts.

### 6.3. Quality by Design (QbD) Implementation and Regulatory Standards

QbD principles link formulation variables and process parameters to final product quality attributes [130,150,151]. For IA PLGA products, Quality Target Product Profiles typically include particle size distribution, drug loading, release kinetics, sterility, and injectability [130,151]. Critical Material Attributes (CMAs) include polymer molecular weight and composition, drug particle size, and excipient properties [152], while Critical Process Parameters (CPPs)—such as emulsification settings, temperature, and solvent removal—are commonly optimized through Design of Experiments (DoE) [146,151,153]. Design Space definition uses statistical methods to map acceptable input–output relationships [154], and sterilization, often via gamma irradiation, requires validation to confirm polymer integrity and release stability [155]. In practice, Zilretta^®^ incorporated Phase II–III scale equivalence within a QbD framework to maintain defined CPPs and CQAs without post-approval process changes [156].

### 6.4. Supply Chain Management and Commercial Considerations

From a cost analysis perspective, the supply of pharmaceutical-grade PLGA remains a key quality determinant, although the number of qualified suppliers has grown in recent years [157]. Standard pharmaceutical supply chain principles apply, with added emphasis on maintaining cold-chain integrity for temperature-sensitive formulations [157]. Manufacturing complexity and quality requirements contribute to higher unit costs compared with conventional injectables [144], making robust pharmacoeconomic evidence essential for payer reimbursement [158,159].

From a market access and policy perspective, payer acceptance hinges on demonstrating therapeutic and economic value supported by well-constructed dossiers and real-world evidence, while post-marketing obligations—such as pharmacovigilance, periodic safety reporting, and, where requested, additional studies—should be integrated into global distribution strategies. Given the extended dosing intervals typical of depot formulations, such integration is important to ensure sustained risk–benefit oversight across markets [157,159,160].

## 7. Clinical Integration and Unresolved Issues in PLGA-Based IA Therapies

### 7.1. Repeat Administration and Safety

PLGA-based IA depots, such as triamcinolone acetonide extended-release (Zilretta^®^), provide prolonged symptom relief and reduce injection frequency compared with conventional IA corticosteroids. In pivotal studies, a single injection maintained clinical benefit for approximately 12 weeks [137]. This extended duration offers a meaningful advantage for patients with chronic joint diseases; however, the safety profile for repeat administration remains incompletely characterized. In a Phase IIIb trial, mild-to-moderate arthralgia was reported in 16% of patients after a second injection compared with 6% after the first [137]. While human imaging studies found no radiographic cartilage damage, preclinical canine models demonstrated transient, dose-related cartilage changes that resolved over time [161]. Taken together, these findings suggest that while repeat dosing is clinically feasible, there is potential for cumulative effects, warranting careful investigation. To address this, dedicated long-term studies—preferably multicenter Phase III trials with extended follow-up periods—should incorporate quantitative imaging, histological correlation where feasible, and patient-reported outcomes to comprehensively evaluate structural and symptomatic changes across repeated dosing cycles.

### 7.2. Clinical Injection Techniques and Considerations

While PLGA microsphere suspensions and conventional intra-articular (IA) corticosteroid solutions are both delivered by direct injection, several critical differences arise from the physical properties of the formulations. Conventional agents like triamcinolone acetonide are low-viscosity solutions (1–3 cP) that can be reliably injected using standard 21–23-gauge needles for large joints and 23–25 gauge for small joints, without concern for clogging or particle settling [162]. PLGA microsphere suspensions, however, contain solid particles (typically 10–100 μm) in more viscous vehicles (20–100 cP), which can settle over time and significantly increase the risk of needle blockage [163]. This often necessitates larger bore needles (e.g., 18–21 gauge for >50 μm particles) and demands prior resuspension by gently rolling the syringe to ensure a uniform dose [164]. For particles >100 μm, an 18-gauge needle is needed to avoid blockage, which is a substantial shift from the technique for corticosteroid solutions [162]. Suspension viscosity must also be well-controlled: too low and particles rapidly settle (causing dosing inconsistency), too high and injection becomes difficult [165]. The injection should be performed soon after resuspension, and the syringe should be oriented vertically while aspirating to ensure homogeneity [166]. These requirements represent a clear departure from the less restrictive protocols for solution formulations.

In summary, the requirement for larger needles, careful resuspension, and monitoring for potential site reactions are all practical considerations unique to PLGA microsphere suspensions, distinguishing them from conventional IA corticosteroid injections [167].

### 7.3. Manufacturing-to-Practice Gap

Bridging clinical evidence and economic feasibility with manufacturing readiness depends on consistent translation of commercial-scale output into predictable in vivo performance. Maintaining tight control over critical product attributes—particle size distribution, drug loading, and release kinetics—is essential, as variations can directly impact pharmacokinetics and pharmacodynamics in the joint. These quality attributes must be preserved across all commercial batches through robust QbD frameworks and integration of PAT to enable real-time monitoring and adjustment during manufacturing. In parallel, supply chain systems must safeguard sterility and, where applicable, cold chain integrity to maintain stability from production to administration. The extended dosing intervals characteristic of depot formulations also add complexity to post-marketing surveillance: adverse events may emerge weeks or months after treatment, making causal attribution more challenging. Addressing this manufacturing-to-practice gap will require coordinated alignment between manufacturing oversight, clinical monitoring, and regulatory review to ensure that marketed products consistently match the safety, efficacy, and release characteristics established in clinical trials.

### 7.4. Unresolved Challenges and Future Directions

Section 7.1, Section 7.2 and Section 7.3 collectively highlight three interdependent barriers to the clinical adoption of PLGA-based IA depots: uncertainty in repeat-dose safety, procedural constraints unique to particulate suspensions, and the manufacturing-to-practice gap linking batch variability to in-joint performance. Addressing these issues demands an integrated framework that unites long-term clinical evaluation with formulation design and process control.

The near-term priorities are fourfold, as summarized in Table 7: (i) establish repeat-administration safety through multicenter trials powered for structural and patient-reported outcomes with ≥2-year follow-up; (ii) standardize injection practice using rheology-guided suspension design, predefined resuspension procedures, and gauge selection appropriate to particle size; (iii) strengthen manufacturing reliability via QbD frameworks and PAT-based real-time CPP/CQA monitoring with secure supply chain management; and (iv) mitigate challenges in biologic formulations by buffering the acidic microenvironment, employing protective excipients, and optimizing copolymer composition supported by validated stability analytics.

Beyond these operational elements, progress will depend on clinical programs that incorporate health-economic endpoints and on proactive regulatory dialog to align evidentiary expectations—topics explored further in Section 8. Collectively, this framework transforms a broad set of unresolved risks into a structured, testable roadmap for translating PLGA-based IA depots into clinically and commercially sustainable therapies.

## 8. Strategic Outlook and Future Development Pathways

The development of PLGA-based sustained-release IA therapies has moved beyond proof-of-concept, demonstrating meaningful clinical benefit in extending symptom relief and reducing injection frequency. These advances have been supported by improved understanding of polymer–drug interactions, scalable manufacturing methods, and early clinical evidence in indications such as knee osteoarthritis. Concurrent advances in molecular imaging and joint physiopathology understanding, including emerging radiotracers for synovial inflammation assessment and deeper insights into disease-specific clearance mechanisms, are providing new opportunities to optimize PLGA-based delivery systems for different stages and types of joint pathology [168,169]. First, the lack of definitive, long-term safety data for repeat administration remains the principal barrier to broad adoption. Existing studies have highlighted changes in adverse event profiles between first and subsequent doses, but robust evidence from multicenter Phase III trials with extended follow-up—at least 2 years (preferably 3 years)—is required to guide labeling expansion and clinical confidence.

Second, advancing formulation technologies is essential to meet the unique demands of biologic therapeutics. While PLGA depots have proven effective for small-molecule drugs, encapsulating proteins or peptides introduces additional challenges in stability, aggregation control, and immunogenicity risk. Targeted innovation should focus on approaches such as co-encapsulation of buffering agents to mitigate local pH drop during polymer degradation, incorporation of protective excipients to preserve molecular integrity, and the use of novel copolymer blends designed for controlled degradation rates and improved biocompatibility. These efforts require validated analytical tools capable of detecting subtle structural changes in biologic payloads during manufacturing, storage, and in situ release.

Third, economic and market access considerations must be addressed explicitly. The higher manufacturing complexity and per-dose cost of sustained-release IA products should be offset by clear pharmacoeconomic value. This, in turn, requires prospective integration of health-economic endpoints into pivotal trials, alongside real-world evidence demonstrating reduced healthcare utilization—such as fewer clinic visits, lower analgesic use, or delayed surgical intervention. Advanced imaging biomarkers and physiopathological understanding will be instrumental in developing personalized treatment approaches that can demonstrate superior value in specific patient populations [168,169]. Early engagement with payers can help align trial designs with outcomes relevant to reimbursement decision-makers, reducing barriers to formulary inclusion.

Finally, regulatory convergence will play an enabling role. While harmonized global standards may be a long-term goal, an achievable first step is bilateral alignment between major agencies—such as the FDA and EMA—on combination product classification, preclinical safety requirements, and pivotal trial design. This alignment could reduce duplication in data packages, shorten approval timelines, and facilitate coordinated global market entry. Achieving such coordination will require structured public–private dialog among regulators, industry, clinicians, and materials scientists to resolve both technical and translational bottlenecks.

By focusing on these interlinked priorities—long-term repeat-dose safety evaluation, innovation in formulation for biologics, and integrated economic plus regulatory strategies—PLGA-based sustained-release IA therapies can progress from niche products to widely adopted treatment options, ultimately improving patient outcomes and the efficiency of musculoskeletal disease management [170,171].

## Figures and Tables

**Table 2:** PLGA-based Intra-articular systems in clinical development.

Product	Drug/Dose	Formulation	Status	Duration	Key Clinical Outcomes
Zilretta^®^	Triamcinolone acetonide 32 mg	75:25 PLGA microspheres (~45 µm)	FDA Approved (2017)	~12 weeks	Superior to immediate-release steroid; repeat dosing safety under evaluation
EP-104IAR	Fluticasone propionate 25 mg	Diffusphere^®^ polymer microspheres	Phase II completed (2022)	~14 weeks	Minimal systemic exposure; Phase III trials planned

**Table 3:** Non-PLGA IA Sustained-Release Products.

Product	Drug/Dose	Technology Platform	Status	Duration	Clinical Highlights
TLC599	Dexamethasone sodium phosphate 12 mg	Multivesicular liposomes (DepoFoam^®^)	Phase III ongoing (Phase II complete)	~24 weeks	Longest duration; well-tolerated in Phase II trials
Cingal^®^	Triamcinolone hexacetonide 18 mg (+ HA 88 mg)	Cross-linked HA composite	Phase III completed (not FDA-approved)	~26 weeks	Dual-action: immediate steroid + sustained HA benefits
Joyclu^®^	Diclofenac-HA conjugate 30 mg	Covalent drug-polymer conjugate	Approved in Japan (2021)	Multi-month	First sustained-release NSAID; rare anaphylaxis risk noted

**Table 4:** PLGA-Based Biologic Therapeutics for IA Delivery.

Agent	Platform	Mechanism	Duration	Status	References
Infliximab (anti-TNF-α mAb)	PLGA microspheres (~45 µm)	TNF-α neutralization	~3 weeks	Advanced preclinical	[89,90]
TNF-α siRNA	PLGA microspheres (~200 nm)	TNF-α gene silencing	>14 days	Proof-of-concept	[91]
IL-1Ra (anakinra)	PLGA microspheres (50:50 copolymer)	IL-1 pathway blockade	4–6 weeks	Preclinical	[92,93,94]
TGF-β1	PLGA microspheres	Chondrogenesis promotion	4–8 weeks	Preclinical	[95,96]
p66shc siRNA	PLGA nanoparticles (~180 nm)	p66shc gene silencing	21 days	Preclinical	[97]

**Table 5:** Biologic Stabilization Strategies in PLGA Systems.

Stabilization Strategy	Target Biologics	Challenge Addressed	Key Mechanism	Outcomes and Clinical Advantages	References
Magnesium hydroxide (Mg(OH)_2_) co-encapsulation	GLP-1 analogs, insulin, growth factors	Neutralizes acidic pH (2–3 → 6.5–7.4)	Acid-base neutralization	Complete release: 50% → >85%; prevents degradation & immunogenicity	[56,102]
Trehalose co-encapsulation	Proteins, antibodies, enzymes	Prevents acid-induced unfolding	Molecular chaperone, hydrogen bonding	>95% activity retention at pH 3; enhanced storage stability	[103,104]
Magnesium carbonate (MgCO_3_) buffering	pH-sensitive biologics, peptides	Sustained pH buffering control	Controlled CO_2_ release mechanism	pH 6.8–7.2 for 8 weeks; reduced inflammation.	[56,102]
Ultrasonic atomization processing	Large proteins, antibodies	Reduces processing-induced aggregation	Gentle processing uniform distribution	3–4× encapsulation efficiency; better batch consistency	[105]

**Table 6:** Regulatory and CMC Workflow for PLGA-Based Intra-Articular Depots.

Step	Key Activities and Deliverables
Pre-RFD Submission	Confirm combination-product classification under 21 CFR 3.2(e); obtain lead center assignment and initial guidance on nonclinical and CMC expectations
Pre-IND Meeting	Finalize IA route-specific toxicology (local tolerability, biodistribution), define QTPP/CQAs, agree on in vitro release methods, and establish trial endpoints.
IND/NDA Submission	Submit IA-focused nonclinical package and CMC dossier including PAT data (FBRM particle sizing; NIR solvent monitoring), polymer specifications, and release profiles.
Phase I–III Clinical Evaluations	Conduct Phase I safety/PK studies; Phase II dose-ranging and preliminary efficacy; Phase III pivotal trials with long follow-up, imaging, and patient-reported outcomes.
NDA/BLA Review & Approval	Provide Module 3 CMC details (polymer Mw/ratio/end-cap, in vitro–in vivo correlation), validation of sterilization/endotoxin methods; undergo pre-approval inspection.
Post-Marketing Surveillance	Implement pharmacovigilance specific to long dosing intervals; conduct device-type postmarket surveillance if required for combination product configuration.

**Table 7:** Concluding Summary of Unresolved Challenges and Future Directions for PLGA-based Intra-Articular Depots.

Domain	Unresolved Challenge	Strategic Direction
Safety & Repeat Dosing	Limited long-term safety data for multiple injections; potential cumulative joint effects	Conduct multicenter Phase III trials with ≥2 years follow-up, incorporating quantitative imaging, histology, and PROs
Injection Technique	Needle blockage, suspension settling, and dosing inconsistency	Optimize suspension rheology; develop standardized resuspension protocols and needle selection guidelines
Biologic Formulation	Protein/peptide instability, aggregation, and immunogenicity in acidic microenvironment	Co-encapsulate buffering excipients; novel copolymer blends; validate analytical methods for real-time stability assays
Manufacturing & Scale-Up	Batch-to-batch variability in particle size, drug loading, and release kinetics; supply chain integrity	Implement QbD with PAT tools for real-time CPP/CQA control; establish robust cold-chain logistics where needed

## Data Availability

No new data were created or analyzed in this study.
